# Longitudinal Profiles and Predictors of Physical Activity in Cancer Survivors Post-Exercise Intervention: A 5-Year Follow-Up of the Phys-Can RCT

**DOI:** 10.1177/15347354251362447

**Published:** 2025-08-08

**Authors:** Anna-Karin Ax, Andreas Stenling, Sveinung Berntsen, Sussanne Börjeson, Karin Nordin, Ingrid Demmelmaier, Anne-Sophie Mazzoni

**Affiliations:** 1 Uppsala University, Sweden; 2 Linköping University, Sweden; 3 Umeå University, Sweden; 4 University of Agder, Kristiansand, Norway

**Keywords:** breast cancer, colorectal cancer, prostate cancer, physical activity profile, physical activity predictors, survivorship, accelerometer

## Abstract

**Background:**

Regular physical activity improves health outcomes in cancer survivors; however, maintaining recommended levels of moderate-to-vigorous intensity physical activity (MVPA) post-treatment is challenging, even for those participating in exercise intervention studies. Understanding long-term MVPA patterns and predictors can guide strategies to promote sustained physical activity in this population. We aimed to describe objectively measured MVPA-profiles over 5 years in cancer survivors who participated in a 6-month exercise intervention during cancer treatment, and to identify baseline predictors of profile belonging.

**Methods:**

Data were derived from the multicenter randomized controlled trial Phys-Can, including participants with breast, colorectal or prostate cancer. Objective measures of MVPA were conducted at baseline, post-intervention, and at 1-, 2-, and 5-year follow-ups. Longitudinal latent profile analysis was used to identify MVPA profiles, and multinomial logistic regression to examine potential baseline predictors of profile belonging.

**Results:**

Among 556 participants, 4 longitudinal MVPA profiles were identified: *Low and stable* (18.0%), Medium *and stable* (40.8%), *High and decreasing* (28.4%), and *Very high and stable* (12.8%). Compared to the *Very high and stable* MVPA profile, participants in the *Low and stable* MVPA profile were more likely to be women (OR = 20.64) or have higher BMI (OR = 1.41) or lower cardiorespiratory fitness (OR = 0.69) at baseline.

**Conclusion:**

Cancer survivors who are women or have a higher BMI and/or low cardiorespiratory fitness prior to cancer treatment are at greater risk of maintaining low MVPA levels over time. These groups may require targeted support to enhance and sustain physical activity during survivorship.

## Background

Regular physical activity is safe and beneficial for cancer survivors.^1^ Systematic reviews have demonstrated positive effects of physical activity on several cancer-related health outcomes, including cancer-related fatigue,^2^ depression^3^ and anxiety,^4^ health-related quality of life (HRQoL),^5^ and physical fitness^6^ both during and after oncological treatment. Additionally, regular physical activity is associated with reduced mortality and lower cancer recurrence.^7^ Consequently, cancer survivors have much to gain from regular physical activity and are therefore recommended to engage in at least 150 minutes of moderate-to-vigorous intensity physical activity (MVPA) per week,^8^ corresponding to physical activities ≥3 metabolic equivalents (METs), similar to the recommendations for the general population. Despite these benefits, many cancer survivors struggle to maintain recommended MVPA levels due to, for example, side effects.^9,10^ Even those participating in exercise interventions during treatment often find it challenging to sustain physical activity levels post-intervention.^11-14^ This highlights the need for a more thorough understanding of how to promote long-term physical activity in cancer survivors.^15,16^

Research on long-term physical activity (≥5 years post-intervention) in cancer survivors is limited,^14,17,18^ and has primarily focused on finding an average pattern of MVPA change over time rather than identifying differences in patterns of change within the population. In addition, the findings regarding factors that influence long-term physical activity are mixed. Physical activity habits^11,17^ and aerobic fitness^13,17^ are considered strong predictors of sustained MVPA over time, whereas the impact on health-related outcomes, such as fatigue, depressive symptoms, and QoL, remains inconclusive.^11-14,17^ Other potential predictors include self-efficacy,^17,19^ motivational support,^13,19^ smoking habits,^11,20^ age,^20^ body mass index,^11,17,20^ and education level.^11,17^ Reliance on self-reported physical activity in previous studies may have led to biased results due to being either overestimated or underestimated.^21^ Therefore, identifying long-term physical activity patterns using objective measures and potential predictors enhances the understanding of different MVPA profiles in cancer survivors post-exercise interventions. This can help to identify cancer survivors at risk of low or declining levels of MVPA. Providing targeted support can help maintain or increase their physical activity level long term. This will aid the design of more effective exercise interventions to promote sustained physical activity among cancer survivors.

In the present study, we aimed to describe objectively measured MVPA profiles over 5 years in cancer survivors who participated in a 6-month exercise intervention during cancer treatment. We also aimed to identify baseline predictors of MVPA profile belonging.

## Method

### Study Design and Participants

This longitudinal study is a secondary analysis based on data from the multicenter randomized controlled trial Phys-Can (Clinical Trials NCT02473003). The Phys-Can RCT was approved by the Regional Ethical Review Board in Uppsala (Dnr 2014/249) and informed consent was obtained from all participants. The Phys-Can RCT used a 2 × 2 factorial design, randomizing 577 participants to 1 of 4 interventions groups: (1) high intensity (HI) exercise, (2) HI exercise with behavior change support, (3) low-to-moderate intensity (LMI) exercise, and (4) LMI exercise with behavior change support. The primary objective of the Phys-Can RCT was to study the effects on fatigue of HI versus LMI exercise with or without behavior change support in patients undergoing (neo)adjuvant oncological treatment. The inclusion procedure, design methods and outcomes have been described in detail elsewhere.^22,23^ In brief, patients ≥18 years, recently diagnosed with breast, prostate or colorectal cancer and scheduled to begin (neo)adjuvant oncological treatment were invited to participate. Exclusion criteria included patients with cognitive or physical disorders, or other diseases that could impact their ability to exercise. Recruitment occurred across three university hospitals in Sweden between March 2015 and April 2018. At the end of the intervention, participants in the HI group had better cardiorespiratory fitness and greater leg strength, and reported lower fatigue, compared to the LMI group, although the differences were modest. No effect of behavior change support on exercise adherence was found during the intervention.^23,24^ However, at 12-month follow-up, the groups randomized to behavior change support maintained their MVPA level to a greater extent compared to the groups without behavior change support.^24^ In the present study, we included all participants from the Phys-Can RCT with MVPA data at baseline and at least 1 follow-up measurement point, that is, the end of the exercise intervention (6 months after baseline), 1 year-, 2 year-, or 5 year-follow-up after the end of the exercise intervention. Given the modest differences and short-term differences observed between the randomized groups in the original trial, we did not expect meaningful group differences over the extended 5-year follow-up. Therefore, all randomization groups were merged for the present study to identify MVPA trajectories.

### Intervention

The 6-month exercise program began at the start of (neo)adjuvant oncological treatment. The program followed a standardized protocol that included both supervised resistance training and home-based endurance training, as previously detailed.^23^ Briefly, the resistance training was performed twice a week in a group at a public gym and comprised 6 machine-based exercises and 4 core exercises. In the HI groups, participants performed 1 weekly session with 3 sets of 6 repetitions maximum (6 RM), and another session with 3 sets of 10 RM. In the LMI groups, participants performed 1 weekly session consisted of 3 sets of 12 repetitions (corresponding to 50% of 6 RM), and the other session consisted of 3 sets of 20 repetitions (corresponding to 50% of 10 RM). For the endurance training, the HI groups engaged in interval training twice a week (20-40 minutes per session) at 80% to 90% of heart rate reserve (HRR) and the LMI groups participated in 150 minutes of weekly endurance activity, maintaining 40% to 50% HRR, in bouts of at least 10 minutes. Participants randomized to behavior change support received face-to-face support from coaches on up to 9 occasions, jointly with resistance training sessions. Support included goal-setting, action planning, problem-solving, self-monitoring, and follow-up prompts. Weekly meetings were held during the first month, then every 4 to 6 weeks based on individual needs. Coaches used structured worksheets to document goals and plans, which were reviewed and revised at each meeting. For example, participants formulated specific home-based endurance training plans (eg, when, where, and how to exercise), and if plans were not followed, barriers were discussed and strategies adjusted. A written relapse prevention plan was developed at the end of the exercise intervention and followed up at 3 and 9 months via face-to-face or telephone sessions.

### Measures

#### Physical Activity

MVPA was assessed at baseline, at the end of the intervention (6 months after baseline), and at 1 year-, 2 year-, and 5 years-follow-ups after the end of the intervention using the validated activity tracker SenseWear armband mini (SWA, BodyMedia Inc, Pittsburgh, PA, USA). The SWA is a tri-axial accelerometer with heat/skin sensors, and has previously been validated in cancer survivors^25^ and healthy adults.^26,27^ Participants wore the SWA on their upper arm (over the triceps) continuously for 7 days at each measurement time-point. To ensure data validity, the SWA needed to be worn for at least 4 days, including 1 weekend day, with a minimum of 80% wear time per day.^28,29^ The Professional 8.1 Software was employed to calculate SWA wear time and minutes spent at various levels of Metabolic Equivalent Task values (METs). A cut-off point of ≥3.0 METs was used to calculate MVPA.^30^ The total time spent in the MVPA levels was determined by calculating the number of minutes on each valid day that met the MVPA criteria.

#### Potential Baseline Predictors of Profile Belonging

Based on prior research,^11,13,17,19,24^ we identified medical variables, self-reported health-related and motivational outcomes, and cardiorespiratory fitness as potential predictors for MVPA over time. The selection of variables was limited by the sample size requirements. Medical history included cancer diagnosis and chemotherapy treatment (Chemotherapy vs No chemotherapy) retrieved from the medical records. Socio-demographic characteristics were self-reported at baseline and included age, sex, comorbidities (No comorbidities vs Comorbidities). Health-related outcomes were self-reported at baseline and included the Global health status/QoL assessed using the subscale of European Organisation for Research and Treatment of Cancer Quality of Life Questionnaire (EORTC QLQ-C30, range 0-100),^31^ and exercise self-efficacy with the 9-item Exercise Barrier Self-Efficacy Scale (EBSS, range 0-10).^32^ Body mass index (BMI, kg/m2) was calculated from participants’ weight (kilogram) divided by their height (m^2^), as measured by the research staff at baseline. BMI was categorized (normal, overweight, and obese) according to WHO standards.^33^ Cardiorespiratory fitness was measured as maximal oxygen uptake (VO_2_max) with gas exchange measurement at baseline. Participants walked or ran up to the point of exhaustion on a treadmill using a modified Balke protocol.^34,35^ All data were collected between March 2015 and November 2023.

### Statistical Analysis

Mplus version 8.9^36^ was used to estimate the longitudinal latent profile analysis (LLPA)^37^ and identify subgroups with different patterns of physical activity over time. The longitudinal physical activity profiles were estimated based on the levels and changes in physical activity from baseline to the 5-year follow-up, allowing means and variances to differ between the profiles.^38^ The robust Full Information Maximum Likelihood (FIML) estimator was used to handle non-normality and missing data in the outcome variable MVPA.

Information criteria and the entropy value, combined with interpretability and theoretical meaningfulness of the profiles, and parsimony, were used to determine the optimal number of longitudinal latent profiles.^37,39^ A model with lower Akaike’s Information Criterion (AIC), Bayesian Information Criterion (BIC), and sample-size adjusted BIC (aBIC) indicate better model fit compared to a model with higher AIC, BIC, and aBIC, whereas a high entropy value indicates a better categorization of people into their respective profiles. Information criteria (ie, AIC, BIC, aBIC) are relative model fit indices used to compare competing models and the model with the lowest value is the preferred model. Entropy, which can range between 0 and 1, indicates overall classification accuracy or separation between the latent profiles. Higher values indicate better class separation and, in a sense, better model fit. Following recommendations in the literature, we did not use the entropy value to determine the optimal number of profiles,^40^ however, it is considered a useful tool to determine classification accuracy.^41^ Models with 1 to 5 latent profiles in were tested and, at each step, the model with *k* number of profiles was compared to a model with *k* + 1 profiles.

Following the selection of the optimal number of profiles, baseline predictors (age, sex, chemotherapy treatment, comorbidities, BMI, cardiorespiratory fitness, quality of life, and exercise self-efficacy) were added to examine whether they could predict profile belonging using multinomial logistic regression analysis via the R3STEP method in Mplus.^42^ In addition to the baseline predictors, we also adjusted the model for behavior change support because those randomized to receive behavior change support in the main trial had higher maintenance of their physical activity levels than those randomized to not receive behavior change support.^43^ Results are presented as odds ratios (OR) and 95% confidence intervals (CI), which describes change in likelihood of membership in a target profile versus a comparison profile with each unit increase in the predictor. An OR above 1.0 indicates that the predictor is associated with increased odds of being a member of the target profile (vs the comparison profile). An OR below 1.0 indicates that the predictor is associated with decreased odds of being a member of the target profile (vs the comparison profile). Under the assumption of missing at random (MAR), multiple imputation was used to handle missing data in the baseline variables (range from 0% to 12% missing data) using a Markov Chain Monte Carlo (MCMC) simulation generating 20 imputed data sets for the analysis.^44^ As a sensitivity analysis, Bayesian-two-part regression modeling was employed to examine predictors of missing MVPA data over time. The 2-part regression model separates the censored outcome variable into 2 parts: (i) a binary part describing the probability of having non-zero missing data points; and (ii) a continuous part for the rest of the distribution (ie, those with non-zero missing data points).

## Results

### Participants

Of the 577 participants in the Phys-Can RCT, 556 (96%) had MVPA data at baseline and at least 1 follow-up measurement point and were therefore included in the present study. The mean age was 58.7 years (SD 12), with 81% being women. Among the participants, 79% were diagnosed with breast cancer, 51% received chemotherapy, and 52% were overweight or obese (see Table 1).

**Table 1.** Sample Characteristics of Participants at Baseline and Distribution of Physical Activity Levels Over 5 Years.

Characteristics	Sample	N
Age, M (SD) range (min-max)	58.7 (12.0) (22-85)	556
Sex, n (%)		
Women	448 (80.6)	556
Men	108 (19.4)	
Living with partner, n (%)	415 (77.6)	535
University education, n (%)	324 (60.0)	540
Working situation, n (%)		
Working (full/part-time)	173 (32.3)	536
On sick leave	187 (34.9)	
Retired	176 (32.8)	
Comorbidities (1 or more), n (%)	284 (58.3)	487
Current exercise habits		
Regular aerobic training, n (%)	177 (37.6)	471
Regular resistance training, n (%)	94 (20.6)	456
BMI, kg/m^2^, n (%)		
Normal weight (BMI 18-24.9)	255 (48.5)	526
Overweight (BMI 25-29.9)	184 (35.0)	
Obese (BMI > 29.9)	87 (16.5)	
Cardiorespiratory fitness (VO_2_max), mL/kg/min, M (SD)	29.8 (7.2)	521
Quality of life (0-100), M (SD)^a^	66.0 (20.4)	541
Exercise self-efficacy (0-10), M (SD)^a^	5.5 (1.8)	536
*Breast cancer*		441
Stage, n (%)^b^		
1	223 (57.8	396
2	149 (37.6)	
3	14 (3.5)	
(Neo)adjuvant oncological treatment, n (%)		
Chemotherapy	266 (64.7)	411
Antibody treatment	77 (23.5)	328
Radiation therapy	182 (41.7)	436
Endocrine therapy	302 (73.5)	411
Prostate cancer		95
Stage *n* (%) ^b^		
1	42 (50.6)	83
2	33 (39.8)	
3	8 (9.6)	
(Neo)adjuvant oncological treatment, n (%)		
Radiation therapy	95 (100)	95
Endocrine therapy	50 (73.5)	68
*Colorectal cancer*		20
Stage, n (%)^b^		
2	5 (26.3)	19
3	14 (73.7)	
(Neo)adjuvant oncological treatment, n (%)		
Chemotherapy	20 (100)	20
MVPA, min/day, M (SD)		
Baseline	72.6 (49.5)	518
6 mo	79.7 (56.4)	418
1-y follow-up	75.6 (50.5)	376
2-y follow-up	72.2 (49.5)	336
5-y follow-up	71.4 (55.5)	255

*Note.* N varies due to missing data, % is of those with data available.

Abbreviations: BMI, body mass index; VO_2_max, maximal oxygen uptake; EORTC QLQ-C30, European Organisation for Research and Treatment of Cancer; EBSS, Exercise Barrier Self-Efficacy Scale; MVPA, moderate-to-vigorous intensity physical activity.

a Higher scores indicate better outcome.

b According to the seventh edition of the Tumour-Node-Metastasis (TNM) clinical classification.^45^

### Longitudinal Profiles of MVPA

We estimated LLPA with 1 to 5 profiles and used information criteria in combination with interpretability, theoretical meaningfulness, and parsimony to determine the optimal number of longitudinal latent profiles. The information criteria (AIC, BIC, and aBIC) decreased substantially with the addition of each profile and reached their lowest point for the 5-class solution (see Table 2). However, 1 profile in the 5-class solution consisted of only 1% (n = 7) of the participants, which suggests that this profile had low power and precision relative to the other larger profiles.^46^ Recommendations in the literature are that without a very strong rationale, profiles with small number of cases (<1.0% of the total sample or less than 25 cases) should be rejected.^47^ Hence, to avoid introducing a high degree of uncertainty, low precision, while also considering parsimony and the risk of low power, we therefore selected a 4-class solution as the final model to be used in subsequent analyses.

**Table 2.** Model Fit Statistics of the Longitudinal Latent Profile Analysis.

Nr of profiles	−LL	#FP	AIC	BIC	aBIC	Entropy	Latent profile proportions %
1	−10 215	10	20 449	20 492	20 460	NA	NA
2	−9680	21	19 403	19 494	19 427	0.78	68/32
3	−9478	32	19 020	19 158	19 057	0.76	42/38/20
**4**	**−9402**	**43**	**18 890**	**19 076**	**18 939**	**0.73**	**18/41/13/28**
5	−9343	54	18 794	19 027	18 855	0.80	34/5/38/1/22

*Note*. Bolds refer to the final model used in the subsequent analyses.

Abbreviations: -LL, log-likelihood; #FP, number of free parameters; AIC, Akaike information criterion; BIC, Bayesian information criterion; aBIC, sample-size adjusted Bayesian information criterion; NA, not applicable.

**Figure 1.**
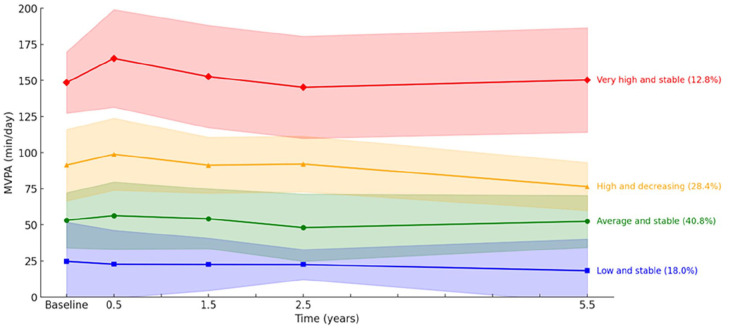
Longitudinal latent moderate-to-vigorous intensity physical activity (MVPA) profiles. Solid lines represent the mean of the MVPA profiles and the colored area represents the respective 95% CIs.

Figure 1 provides a graphical description of the 4 longitudinal MVPA profiles. The *Low and stable* MVPA profile consisted of 18.0% of the participants and was characterized by low initial levels of MVPA that were maintained over time. The *Medium and stable* MVPA profile was the largest and consisted of 40.8% of the participants. Participants in this profile also had a stable pattern of MVPA over time, but on average engaged in almost twice as much MVPA per day compared to the *Low and stable* profile. The *High and decreasing* MVPA profile consisted of 28.4% of the participants who were engaged in high levels of MVPA which on average decreased over time (average decrease from baseline to 5 years ≈ 15 minutes/day). Finally, the *Very high and stable* MVPA profile consisted of 12.8% of the participants who were engaged in very high levels of MVPA. Participants in this profile increased their MVPA from baseline to 6 months (average increase ≈ 17 minutes/day) and then decreased and maintained their baseline levels. In summary, the 4 longitudinal latent profiles differed primarily regarding levels of MVPA, but we also noted differences in the shape of the longitudinal profiles. For example, in the *High and decreasing* MVPA profile, the number of minutes of MVPA per day decreased after a 2-year follow-up, in contrast to the stable MVPA levels observed in the *Very high and stable* MVPA profile. Distribution of background characteristics by MVPA profile is presented in Table 3.

**Table 3.** Distribution of Background Characteristics at Baseline by MVPA Profile Belonging.

Characteristics	MVPA profiles			
	Low and stable (n = 100; 18.0%)	Medium and stable (n = 227; 40.8%)	High and decreasing (n = 158; 28.4%)	Very high and stable (n = 71; 12.8%)
Age, M (SD)	62.5 (11.7)	60.0 (11.8)	55.9 (12.4)	55.5 (10.0)
Sex, n (%)				
Women	87 (87.0)	185 (81.5)	129 (81.6)	47 (66.2)
Men	13 (13.0)	42 (18.5)	42 (18.5)	24 (33.8)
Living with a partner, n (%)	72 (75.8)	164 (74.2)	123 (82.0)	56 (81.2)
University education, n (%)	49 (50.5)	138 (63.0)	107 (69.5)	30 (42.9)
Working situation, n (%)				
Working (full/part-time)	24 (25.3)	70 (32.0)	50 (32.5)	29 (42.6)
On sick leave	27 (28.4)	73 (33.3)	63 (40.9)	24 (35.3)
Retired	44 (46.3)	76 (34.7)	41 (26.6)	15 (22.1)
Comorbidities (1 or more), n (%)	63 (73.3)	121 (60.5)	66 (48.9)	34 (51.5)
Current exercise habits				
Regular aerobic training, n (%)	8 (9.9)	66 (33.5)	72 (53.3)	31 (53.4)
Regular resistance training, n (%)	4 (5.3)	33 (17.4)	38 (28.6)	19 (33.3)
BMI, kg/m^2^, n (%)				
Normal weight (BMI 18-24.9)	18 (19.1)	95 (43.8)	99 (67.3)	43 (63.2)
Overweight (BMI 25-29.9)	26 (27.7)	90 (41.5)	45 (30.6)	23 (33.8)
Obese (BMI > 29.9)	50 (53.2)	32 (14.7)	3 (2.0)	2 (2.9)
Cardiorespiratory fitness (VO_2_max, mL/kg/min, M (SD))	23.2 (4.7)	28.4 (5.6)	33.5 (6.5)	34.9 (7.7)
MVPA min/week, M (SD)	22.3 (12.1)	53.9 (19.4)	94.5 (30.1)	156.1 (55.3)
Global health/QoL (EORTC QLQ C30, 0-100), M (SD)^a^	62.2 (21.8)	66.4 (19.9)	67.7 (19.8)	66.3 (20.8)
Exercise self-efficacy (EBSS, 0-10), M (SD)^a^	4.9 (1.9)	5.5 (1.8)	5.9 (1.7)	5.9 (1.7)
*Breast cancer*				
Stage, n (%)^b^				
1	36 (48.6	111 (66.5)	65 (56.5)	21 (52.5)
2	36 (48.6)	52 (31.1)	42 (36.5)	19 (47.5)
3	2 (2.7)	4 (2.4)	8 (7.0)	
(Neo)adjuvant oncological treatment, n (%)				
Chemotherapy	43 (55.8)	107 (61.1)	81 (69.2)	35 (83.3)
Antibody treatment	13 (22.4)	32 (24.4)	25 (24.5)	7 (18.9)
Radiation therapy	20 (23.5)	66 (36.3)	66 (53.7)	30 (65.2)
Endocrine therapy	59 (76.6)	138 (78.9)	77 (65.8)	28 (66.7)
*Prostate cancer*				
Stage, n (%)^b^				
1	3 (33.3)	22 (62.9)	9 (40.9)	8 (47.1)
2	6 (66.7)	28 (22.9)	11 (50.0)	8 (47.1)
3	0	5 (14.3)	2 (9.1)	1 (5.9)
(Neo)adjuvant oncological treatment, n (%)				
Radiation therapy	12 (100)	37 (100)	25 (100)	21 (100)
Endocrine therapy	7 (22.2)	20 (83.3)	11 (61.1)	12 (70.6)
*Colorectal cancer*				
Stage, n (%)^b^				
2	2 (100)	0	3 (42.9)	2 (50.0)
3	0	6 (100)	4 (57.1)	2 (50.0)
(Neo)adjuvant oncological treatment, n (%)				
Chemotherapy	2 (100)	6 (100)	8 (100)	4 (100)

*Note*. N varies due to missing data, % is of those with data available.

Abbreviations: MVPA, moderate-to-vigorous intensity physical activity; BMI, body mass index; VO_2_max, maximal oxygen uptake; EORTC QLQ-C30; European Organisation for Research and Treatment of Cancer, EBSS; Exercise Barrier Self-Efficacy Scale.

a Higher scores indicate better outcome.

b According to the seventh edition of the Tumour-Node-Metastasis (TNM) clinical classification.^45^

### Predictors of Profile Belonging

The results from the multinomial logistic regression analysis are presented in Table 4, showing relationships between the potential baseline predictors and the longitudinal latent MVPA profiles. The *Very high and stable* MVPA profile was used as the reference profile in the analysis. The results indicate that women were more likely to belong to *Low and stable, Medium and stable*, and *High and decreasing* MVPA profiles compared to the *Very high and stable* MVPA profile. However, the large ORs and the wide CIs suggest that the estimates are uncertain, potentially due to sparse data. Higher BMI at baseline predicted an increased odds (OR = 1.41, 95% CI [1.16-1.71]) of belonging to the *Low and stable* MVPA profile compared to the *Very high and stable* MVPA profile. Higher cardiorespiratory fitness at baseline predicted decreased odds (OR = 0.69, 95% CI [0.57-0.84]) of belonging to the *Low and stable* MVPA profile and a decreased odds (OR = 0.87, 95% CI [0.81-0.93]) of belonging to the *Medium and stable* MVPA profile compared to the *Very high and stable* MVPA profile.

**Table 4.** Multinominal Logistic Regression Predicting MVPA Profile.

Predictor	Low and stable versus very high and stable			Medium and stable versus very high and stable			High and decreasing versus very high and stable		
	OR	95% CI		OR	95% CI		OR	95% CI	
		Lower	Upper		Lower	Upper		Lower	Upper
Age	1.05	0.98	1.13	1.03	0.99	1.07	1.01	0.97	1.06
Sex (women)	**20.64**	**4.66**	**91.32**	**6.03**	**2.25**	**16.14**	**4.63**	**1.45**	**14.81**
Chemotherapy treatment (Yes)	0.78	0.20	2.97	0.66	0.27	1.61	0.65	0.24	1.79
Comorbidities, one or more (Yes)	1.54	0.42	5.70	1.18	0.58	2.40	0.91	0.42	1.97
BMI	**1.41**	**1.16**	**1.71**	1.07	0.96	1.18	0.95	0.84	1.07
Cardiorespiratory fitness	**0.69**	**0.57**	**0.84**	**0.87**	**0.81**	**0.93**	0.96	0.90	1.03
Quality of life	0.99	0.96	1.02	1.00	0.98	1.02	1.01	0.99	1.03
Exercise self-efficacy	0.79	0.56	1.11	0.91	0.74	1.11	1.02	0.81	1.29

*Note.* Statistically significant odds ratio (the 95% CI did not include 0). The model was adjusted for Behavior change support. The Very high and stable MVPA profile was used as the reference profile (coded as 0). Bold indicates *p*-value < .05.

Abbreviations: OR, odds ratios; CI, confidence interval; BMI, body mass index; MVPA, moderate-to-vigorous intensity physical activity.

### Sensitivity Analysis

A Bayesian 2-part regression model^48^ was used to examine the pattern of missing MVPA data over time and to account for missing data points. Higher BMI at baseline predicted higher odds of having 1 or more missing occasions of MVPA, whereas higher exercise self-efficacy at baseline predicted lower odds of having 1 or more missing occasions of MVPA. Additionally, higher VO_2_max at baseline predicted fewer missing occasions of MVPA; however, the effect was relatively weak. The weak effect of baseline MVPA on missing data, and that other baseline predictors (BMI, exercise self-efficacy, and VO_2_max) predicted missing MVPA at subsequent measurement points, strengthens the assumption that the data are missing at random and supports the use of FIML to account for missing MVPA data (see Supplemental Table 1).

## Discussion

We identified 4 different longitudinal MVPA profiles among cancer survivors post-exercise intervention, characterized by *Low and stable* (18.0%), *Medium and stable* (40.8%), *High and decreasing* (28.4%), and *Very high and stable* (12.8%) MPVA patterns. Baseline cardiorespiratory fitness, BMI and sex were significant predictors of MVPA profile belonging. Participants belonging to the *Low and stable* MVPA profile were more likely to be women, have lower cardiorespiratory fitness, and have a higher BMI compared to participants in the *Very high and stable* profile.

The number of MVPA profiles identified in our study contrasts with previous cohort studies that identified 3 trajectories: low, medium, and high MVPA among cancer survivors with up to 2 years of follow-up.^49,50^ Additionally, in our study, only 18.0% of the participants were in the Low and stable profile, which includes individuals who do not meet the recommendation of 150 minutes of MVPA per week.^8^ This proportion is lower compared to 58%^49^ and 42.5%^50^ reported in previous studies. These discrepancies may be attributed to differences in study samples, treatment timeframes, and follow-up periods. In addition, our participants were relatively healthy, and highly motivated to engage in physical activity and were involved in a structured exercise intervention, which may also contribute to the differences in results. At the 6-month follow-up (ie, immediately post-intervention), participants in the high MVPA profiles increased their MVPA levels, while those in the *Low and stable* MVPA profile reverted to their baseline levels of MVPA. These findings suggest that exercise interventions can temporarily increase physical activity levels among already active survivors. However, the long-term benefits of these interventions may diminish over time, highlighting the importance of maintaining established physical activity habits. However, nearly one-fifth of the participants belonged to the *Low and stable* MVPA profile, indicating a need for post-intervention support for less active individuals. Over time, the MVPA profiles stabilized, aligning with patterns observed in previous studies.^49,50^ Such patterns of stability highlight the importance of early identification and targeted interventions for individuals with low physical activity levels.

Participants with a high BMI were more likely to be in the *Low and stable* profile compared to the *Very high and stable profile*, which is consistent with previous studies.^51,52^ Similarly, participants with lower cardiorespiratory fitness were more likely to be in the *Low and stable* or the *Medium stable* MVPA profiles compared to the *Very high and stable* MVPA profile. These findings reinforce the relationship between physical fitness and sustained physical activity levels over time, as reported in another exercise study involving breast cancer survivors.^17^ Furthermore, our results showed that women were more likely to belong to the *Low and stable*, *Medium stable* or *High and decreasing* profiles than to the *Very high and stable* MVPA profile, although this finding should be interpreted with caution due to uncertain estimates. QoL and comorbidities were not identified as predictors of MVPA profile belonging, similar to one of the previously mentioned studies.^50^ In contrast to one previous study,^52^ we did not find self-efficacy to predict profile membership. A possible explanation for this discrepancy is the relatively limited variation in self-efficacy in our sample, or the much longer follow-up period in our study. Notably, most previous studies have assessed self-efficacy in relation to short-term physical activity outcomes (eg, adoption or 6-12 month maintenance). Indeed, several researchers have argued that predictors of physical activity behavior may vary across the different phases of the behavior change process, such as initiation, adoption, and short- versus long-term maintenance.^53,54^ For example, factors like exercise self-efficacy may be particularly important during the initiation or early adoption phases, but may not play the same role in sustaining behavior over the long term.^55^ This highlights the importance of considering the timing and context when examining psychosocial predictors of physical activity behavior.

Our findings highlight the importance of identifying cancer survivors at risk of low MVPA levels during cancer survivorship, particularly at the start of treatment and even after taking part in comprehensive exercise interventions. Targeted interventions should focus on motivating survivors who are overweight or obese and/or have poor cardiorespiratory fitness, since they are more likely to have lower MVPA-levels during their cancer survivorship and require additional support to increase their physical activity levels. Such support could include the use of behavior change techniques, such as self-monitoring and goal-setting, which have been proven to be effective in promoting long-term physical activity post-intervention in cancer populations.^16^ Future research should prioritize interventions that not only motivate physically inactive survivors to engage in physical activity during treatment, but also support sustained adherence to physical activity recommendations throughout survivorship.

### Strengths and Limitations

The strengths of this study include its longitudinal design with long-term follow-ups. To our knowledge, this is the first study to use objective physical activity measures to identify distinct longitudinal MVPA profiles and their predictors over a 5-year period in cancer survivors. The inclusion of objective measures of physical activity adds robustness to the findings, providing valuable insights into long-term patterns and predictors of MVPA. The 4 longitudinal profiles of MVPA identified in this study were determined using LLPA, which is a model-based approach that accounts for uncertainty in the classification of individuals to different profiles, both in the profile estimation and when predictors of the profiles are included. Furthermore, being able to rely on both statistical criteria and substantive judgment when deciding on the number of profiles makes LLPA a less subjective and more robust approach for person-centered analysis. However, the study has limitations. Due to sample size constraints, we examined a limited number of predictors, which may have restricted the identification of additional predictors for MVPA over time. MVPA data was missing for 40% participants at 2 years and for 46% participants at 5 years follow up. Although our sensitivity analysis did not indicate systematic missingness due to MVPA levels, the amount of missingness may introduce uncertainty in the estimation. MVPA was measured at 5 time points, with a 3-year gap between the last 2 measurements. Consequently, it is possible that not all changes in MVPA levels were captured over time. Additionally, although the SWA used to measure MVPA is a validated multi-sensor device that incorporates 3-axis accelerometry to improve accuracy and reduce the risk of overestimation, some degree of overestimation, particularly for certain types of activities such as walking and ergocycling, cannot be ruled out.^25^ Furthermore, the homogeneity of the study sample (ie, predominantly highly educated, relatively healthy and physically active women with breast cancer) may also limit the generalizability of our findings. Finally, we did not distinguish between different types of physical activity in the current study, which could have provided a more nuanced understanding of changes in physical activity patterns over time. Despite these limitations, the study provides valuable insights into long-term physical activity patterns and predictors post-exercise intervention in cancer survivors.

## Conclusions

This study identified 4 distinct MVPA profiles among cancer survivors who participated in an exercise intervention during cancer treatment, revealing the variability in long-term physical activity trajectories over a 5-year period. Most participants maintained stable physical activity levels over time, with only 18.0% having low MVPA and 12.8% achieving very high levels of physical activity. Higher BMI and/or lower cardiorespiratory fitness at baseline, and being a woman were associated with lower MVPA profiles, suggesting that these specific groups may need extra attention and support. However, estimates for sex were large and should therefore be interpreted with caution. These findings emphasize the importance of early identification of individuals at risk, and targeted strategies to promote and sustain physical activity in cancer survivors, even among those participating in comprehensive exercise interventions.

## Supplemental Material

sj-docx-1-ict-10.1177_15347354251362447 – Supplemental material for Longitudinal Profiles and Predictors of Physical Activity in Cancer Survivors Post-Exercise Intervention: A 5-Year Follow-Up of the Phys-Can RCT Supplemental material, sj-docx-1-ict-10.1177_15347354251362447 for Longitudinal Profiles and Predictors of Physical Activity in Cancer Survivors Post-Exercise Intervention: A 5-Year Follow-Up of the Phys-Can RCT by Anna-Karin Ax, Andreas Stenling, Sveinung Berntsen, Sussanne Börjeson, Karin Nordin, Ingrid Demmelmaier and Anne-Sophie Mazzoni in Integrative Cancer Therapies
