# Supplementary material for: Longitudinal Profiles and Predictors of Physical Activity in Cancer Survivors Post-Exercise Intervention: A 5-Year Follow-Up of the Phys-Can RCT
Source: Integr Cancer Ther. 2025 Aug 8;24:15347354251362447. doi: 10.1177/15347354251362447 (PMC12334827; doi:10.1177/15347354251362447)
Supplement: sj-docx-1-ict-10.1177_15347354251362447 – Supplemental material for Longitudinal Profiles and Predictors of Physical Activity in Cancer Survivors Post-Exercise Intervention: A 5-Year Follow-Up of the Phys-Can RCT [file sj-docx-1-ict-10.1177_15347354251362447.docx]

**Supplementary Table 1**. Bayesian two-part regression analysis predicting number of missing occasions of Moderate-to-vigorous intensity physical activity (MVPA)

|  |  | Binary part | |  |  | Continuous part | |
| --- | --- | --- | --- | --- | --- | --- | --- |
|  |  | 95%  credibility interval | |  |  | 95%  credibility interval | |
| **Predictor** | ***OR*** | ***LL*** | ***UL*** | ***b*** | ***SD*** | ***LL*** | ***UL*** |
| Behavior change support (Yes) | 0.97 | 0.68 | 1.39 | -0.01 | 0.06 | -0.12 | 0.11 |
| Age | 1.00 | 0.98 | 1.02 | -0.01 | 0.00 | -0.01 | 0.00 |
| Sex (Women) | 0.98 | 0.56 | 1.69 | 0.01 | 0.09 | -0.17 | 0.19 |
| Chemotherapy treatment (Yes) | 1.33 | 0.86 | 2.06 | 0.01 | 0.08 | -0.14 | 0.16 |
| Comorbidities, one or more (Yes) | 1.28 | 0.85 | 1.92 | 0.02 | 0.07 | -0.12 | 0.15 |
| BMI | **1.06** | **1.00^a^** | **1.12** | -0.01 | 0.01 | -0.03 | 0.01 |
| Cardiorespiratory fitness | 1.00 | 0.96 | 1.04 | **-0.02** | **0.01** | **-0.03** | **-0.01** |
| Quality of life | 1.00 | 0.99 | 1.01 | 0.00 | 0.00 | -0.01 | 0.00 |
| Exercise self-efficacy | **0.90** | **0.81** | **1.00^b^** | -0.02 | 0.02 | -0.05 | 0.02 |
| Baseline MVPA | 1.00 | 1.00 | 1.01 | 0.00 | 0.00 | 0.00 | 0.00 |

Abbreviations: *OR*, odds ratio; *b*, unstandardized regression coefficient; *SD*, posterior standard deviation; *LL*, lower limit, *UL,* upper limit; BMI, body mass index. Statistically significant effects are in bold. Note: a LL = 1.001; b UL = 0.998. The model was adjusted for Behavior change support.

Interpretation of Supplementary Table 1: For the binary part, ORs refer to the odds of having one or more missing occasions of MVPA and indicates that higher BMI at baseline predicts higher odds of having one or more missing occasions of MVPA, whereas higher exercise self-efficacy at baseline predicts lower odds of having one or more missing occasions of MVPA. For the continuous part, the unstandardized regression coefficients *(b)* refer to the effect of a one-unit increase in the baseline predictors on missing occasions of MVPA among those with one or more missing occasions. The results show that higher VO2max at baseline predicts fewer missing occasions of MVPA; however, the effect is relatively weak.
